# Supplementary material for: Causal Relationship between Aging and Anorexia Nervosa: A White-Matter-Microstructure-Mediated Mendelian Randomization Analysis
Source: Biomedicines. 2024 Aug 16;12(8):1874. doi: 10.3390/biomedicines12081874 (PMC11351342; doi:10.3390/biomedicines12081874)
Supplement: Supplementary file 1 [file biomedicines-12-01874-s001.zip › Supplementary Figure/Figure S4/Singe SNP plot of MR analysis for TL to ubm-b-2093.pdf]

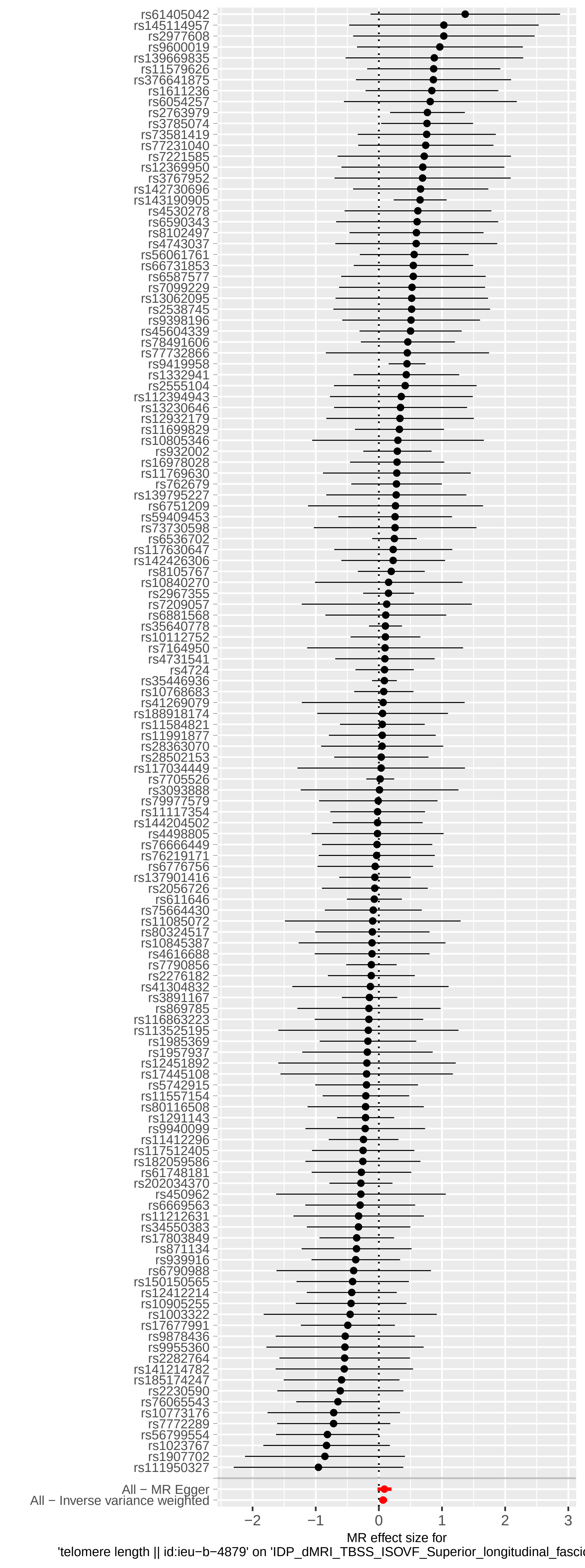
rs61405042
rs145114957
rs2977608
rs9600019
rs139669835
rs11579626
rs376641875
rs1611236
rs6054257
rs2763979
rs3785074
rs73581419
rs77231040
rs7221585
rs12369950
rs3767952
rs142730696
rs143190905
rs4530278
rs6590343
rs8102497
rs4743037
rs56061761
rs66731853
rs6587577
rs7099229
rs13062095
rs2538745
rs9398196
rs45604339
rs78491606
rs77732866
rs9419958
rs1332941
rs2555104
rs112394943
rs13230646
rs12932179
rs11699829
rs10805346
rs932002
rs16978028
rs11769630
rs762679
rs139795227
rs6751209
rs59409453
rs73730598
rs6536702
rs117630647
rs142426306
rs8105767
rs10840270
rs2967355
rs7209057
rs6881568
rs35640778
rs10112752
rs7164950
rs4731541
rs4724
rs35446936
rs10768683
rs41269079
rs188918174
rs11584821
rs11991877
rs28363070
rs28502153
rs117034449
rs7705526
rs3093888
rs79977579
rs11117354
rs144204502
rs4498805
rs76666449
rs76219171
rs6776756
rs137901416
rs2056726
rs611646
rs75664430
rs11085072
rs80324517
rs10845387
rs4616688
rs7790856
rs2276182
rs41304832
rs3891167
rs869785
rs116863223
rs113525195
rs1985369
rs1957937
rs12451892
rs17445108
rs5742915
rs11557154
rs80116508
rs1291143
rs9940099
rs11412296
rs117512405
rs182059586
rs61748181
rs202034370
rs450962
rs6669563
rs11212631
rs34550383
rs17803849
rs871134
rs939916
rs6790988
rs150150565
rs12412214
rs10905255
rs1003322
rs17677991
rs9878436
rs9955360
rs2282764
rs141214782
rs185174247
rs2230590
rs76065543
rs10773176
rs7772289
rs56799554
rs1023767
rs1907702
rs111950327
All − MR Egger
All − Inverse variance weighted
−2
−1
0
1
2
3
MR effect size for
'telomere length || id:ieu−b−4879' on 'IDP_dMRI_TBSS_ISOVF_Superior_longitudinal_fascic
